# Auditory Cortical Plasticity in Patients with Single-Sided Deafness Before and After Cochlear Implantation

**DOI:** 10.1007/s10162-024-00928-3

**Published:** 2024-01-22

**Authors:** Nicole Peter, Valerie Treyer, Rudolf Probst, Tobias Kleinjung

**Affiliations:** 1 Department of Otorhinolaryngology, Head & Neck Surgery, University Hospital Zurich, University of Zurich, Rämistrasse 100, CH-8091 Zurich, Switzerland; 2 Department of Nuclear Medicine, University Hospital Zurich, University of Zurich, Zurich, Switzerland; 3 Institute for Regenerative Medicine, University of Zurich, Zurich, Switzerland

**Keywords:** H215O, Cerebral blood flow, Unilateral hearing loss, CI, SSD

## Abstract

**Purpose:**

This study investigated neuroplastic changes induced by postlingual single-sided deafness (SSD) and the effects of a cochlear implantation for the deaf ear. Neural processing of acoustic signals from the normal hearing ear to the brain was studied before and after implantation using a positron emission tomography (PET)/CT scanner.

**Methods:**

Eight patients with postlingual SSD received a cochlear implant (CI) in a prospective clinical trial. Dynamic imaging was performed in a PET/CT scanner using radioactively labeled water ([15O]H2O) to localize changes in the regional cerebral blood flow (rCBF) with and without an auditory task of logatomes containing speech-like elements without meaningful context. The normal hearing ear was stimulated before implantation and after the use of the cochlear implant for at least 8 months (mean 13.5, range 8.1–26.6). Eight age- and gender-matched subjects with normal hearing on both sides served as healthy control subjects (HCS).

**Results:**

When the normal hearing ear of SSD patients was stimulated before CI implantation, the [15O]H2O-PET showed a more symmetrical rCBF in the auditory regions of both hemispheres in comparison to the HCS. The use of CI increased the asymmetry index (AI) in six of eight patients indicating an increase of activity of the contralateral hemisphere. Non-parametric statistics revealed a significant difference in the AI between patients before CI implantation and HCS (*p* < .01), which disappeared after CI implantation (*p* = .195).

**Conclusion:**

The functional neuroimaging data showed a tendency towards normalization of neuronal activity after CI implantation, which supports the effectiveness of CI in SSD patients.

**Trial Registration:**

ClinicalTrials.gov Identifier: NCT01749592, December 13, 2012.

## Introduction

Single-sided deafness (SSD) is defined as severe hearing loss in one ear and normal hearing in the other ear. By consensus, the mean pure-tone hearing threshold (averaged over 0.5, 1, 2, and 4 kHz) in the poorer hearing ear should be at least 70 dB HL, and hearing loss in the better hearing ear should not be more than 30 dB HL [1].

Postlingual SSD leads to neuroplastic changes in the central nervous system (see review of Vanderauwera et al. [2]) including reshaping of the lateralized neural network for auditory processing of the single hearing ear. Such changes can be demonstrated using functional magnetic resonance imaging (fMRI) [2–11], electroencephalography (EEG) [12, 13], or magnetoencephalography (MEG) [14, 15]. Monoaural acoustic stimulation in normal hearing individuals leads to increased cerebral blood oxygen level dependent (BOLD) responses in the contralateral hemisphere [5], and it exhibits hemispheric asymmetry [6]. The processing of speech signals occurs predominantly in the left auditory cortex (AC) [7, 9, 10].

Cochlear implants (CIs) are used to regain hearing in deaf ears by direct electrical stimulation of the auditory nerve through a surgically implanted intracochlear electrode. Van de Heyning et al. began implanting patients with SSD in 2008 [16], focusing primarily on therapy of tinnitus. Many studies have since confirmed the effectiveness of CI as a therapy for tinnitus and for hearing rehabilitation in patients with SSD (e.g., [17–25]).

Functional MRI examinations are difficult to perform in subjects wearing CI, mainly due to artifacts induced by the implanted magnet. For this reason, neuronal plasticity in SSD patients with CI has been mostly investigated using averaged EEG recordings such as cortical auditory evoked potentials (CEAPs) [26–29] or event-related potentials (ERPs) [30]. Another possibility to examine central neuroplasticity without interference from the magnet of CI is positron emission tomography (PET). In this imaging technique, a radionuclide is synthetically introduced into a molecule of biological relevance and administered to the patient. The subsequent brain uptake of the radiotracer is measured over time and used to obtain information about the process of interest. A radiotracer for neuroplasticity studies of the auditory system is radioactively labeled water ([15O]H2O)) [31], which can be used to localize changes in the regional cerebral blood flow (rCBF) as an indirect indication of regional brain activity. Radioactively labeled water ([15O]H2O)) can show rapid changes in brain activity following specific auditory tasks. An advantage of the PET/CT technique over EEG is the better spatial resolution and, thus, more precise localization of the change in neuronal activity.

A recently published study [32] investigated cortical changes in patients with CI due to asymmetric hearing loss using PET ([15O]H2O)). The study demonstrated that the central contralateral dominance during stimulation of the non-implanted ear could be restored with the use of CI. In addition, a correlation of contralateral dominance with sound localization performance was shown. We investigated in a prospective study changes in rCBF in SSD patients before and after CI implantation also using [15O]H2O-PET with an auditory task. The aim of our study was to demonstrate neuroplastic changes due to CI in SSD in comparison with rCBF changes of normal hearing controls. Our focus was on individual and group activation during auditory stimulation and changes in the symmetry of brain activity of single ear stimulation. We assumed that the brain activation due to stimulation of the healthy ear would normalize with the use of CI and that the activation brain patterns should become closer to those of normal hearing controls.

## Materials and Methods

### Study Design and Participants

Five patients with left and five patients with right SSD were enrolled sequentially in this prospective, open, non-randomized clinical trial. The clinical results of these ten patients together with inclusion and exclusion criteria have been published elsewhere [21]. In short, patients had to fulfil the following inclusion criteria: age between 18 and 70 years; acquired SSD due to cochlear damage (hearing loss of ≥ 70 dB HL in the mean thresholds of 0.5, 1, 2, and 4 kHz in the affected ear, and 25 dB HL or better in the frequencies from 125 to 2 kHz and 35 dB HL or better from 4 to 8 kHz in the normally hearing contralateral ear); normal imaging of the cochlea and the cochlear nerve on magnetic resonance imaging (MRI); onset of SSD within 6 months to 10 years before study inclusion; right handedness; impairment of daily life as a consequence of SSD; and unsatisfactory benefit from therapy with a conventional acoustic hearing system which transmits acoustic signals from the deaf ear to the healthy ear via bone conduction (bone-anchored hearing aid, BAHA) or wireless transmission (contralateral routing of signals, CROS) [21]. Patients were excluded if they had any middle ear pathology, psychiatric comorbidity, or severe coexisting illness [21].

**Fig. 1:**
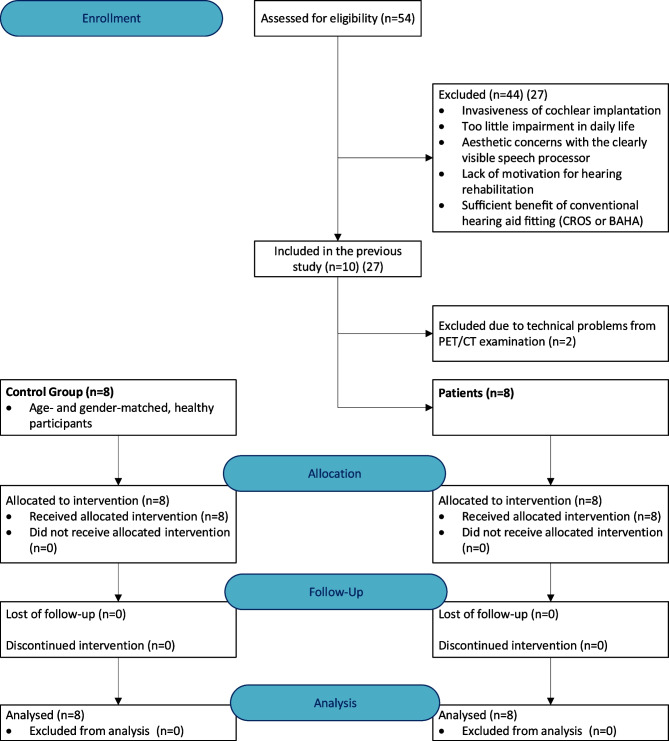
Flow diagram of the progress based on the consort 2010 flow diagram for randomized trials

Patients received a CI on the deaf side. The number of ten subjects was set by cochlear implants provided by the Cochlear Company for this study. At the time of the study, cochlear implants were not reimbursed by healthcare insurances in Switzerland for SSD. Due to unforeseen prolonged technical problems with radionuclide production, PET/CT examination could not be performed in the last two subjects within a useful timeframe. Thus, these two patients were not included in this report (Fig. 1). Results of the PET scans of the eight patients were compared to a control group of eight age- and gender-matched, healthy participants with normal hearing on both sides (HCS: hearing control subjects).

All study participants gave written informed consent before participating. The study was approved by the local institutional review boards (reference numbers KEK-ZH 2012–0034 and KEKBE 233/12).

### [15O]H2O-PET

Dynamic imaging was performed in a PET/CT scanner using [15O]H2O as in previous behavioral studies [33], including neuroplasticity studies of the auditory system [31, 32]. Catheters were placed in the right antecubital vein for tracer injection. Subjects were scanned in a whole-body PET/CT scanner (Discovery RX and DMI; GE Healthcare, Milwaukee, WI) in 3D mode dynamically with 10-s frames over 3 min and with an axial field of view of 14.6 cm. Voxel sizes of 2.34*2.34*3.27 mm were reconstructed with filtered back projection algorithm or SharpIR for DMI Scanner. A low-dose computed tomography was acquired before tracer injection for attenuation correction.

To quantify the dynamically acquired images we used the k2 method without arterial blood sampling [34].

The patients received a total of 12 [15O]H2O-injections containing each approximately 400 MBq. Two sessions of six PET scans, one before and one after CI implantation, were performed in SSD patients, while control participants had only one session. One session included three baseline scans without stimulation and three scans with auditory stimulation. The scans with and without auditory stimulation were recorded alternately using a fixed order of baseline (B)1 – stimulation (ST)1 – B2 – ST2 – B3 – ST3. The normal hearing ear was stimulated by insert-earphone using a sound file with logatomes containing speech-like elements without meaningful content (e.g., apa-du-di-dü-aba…). Duration was 3 min and level 60 dB SL. The PET scans during stimulation were compared to the baseline PET scans to detect stimulation-related changes in brain activity.

Normal HCS had one session with the same experimental design of six scans. They heard the same sound file to the matched ear.

### Analysis

Images were processed with PMOD 3.8 (Pmod Technologies Inc., Zurich Switzerland). Perfusion images (K1) were calculated according to previously described methods [34]. For this processing, we used a Gaussian 5-mm filter to reduce noise. Pre- and postoperative images were averaged separately to receive one average image of each condition (baseline and stimulation) per subject. A normalization procedure was applied to bring all images into a common space. The images per participant were averaged to receive one image to calculate the normalization matrix of each participant brain space. The MNI (Montreal Neurological Institute) template brain was estimated and applied to all individual images.

Images were then exported in NIfTI (Neuroimaging Informatics Technology Initiative) format and further processed with SPM12 (Wellcome Trust Centre for Neuroimaging, London, UK) for statistical parametric mapping (SPM). A Gaussian filter of 16 mm was applied before statistical comparisons were made. Due to the small number of participants, only simple comparisons were performed to test effects of condition and intervention separately. To estimate perfusion changes under acoustic stimulation, we performed a within-subject ANOVA using the K1 images with grand mean scaling to 50 and ANCOVA based global normalization to reduce the variability of the K1 values within and between subjects.

**Fig. 2:**
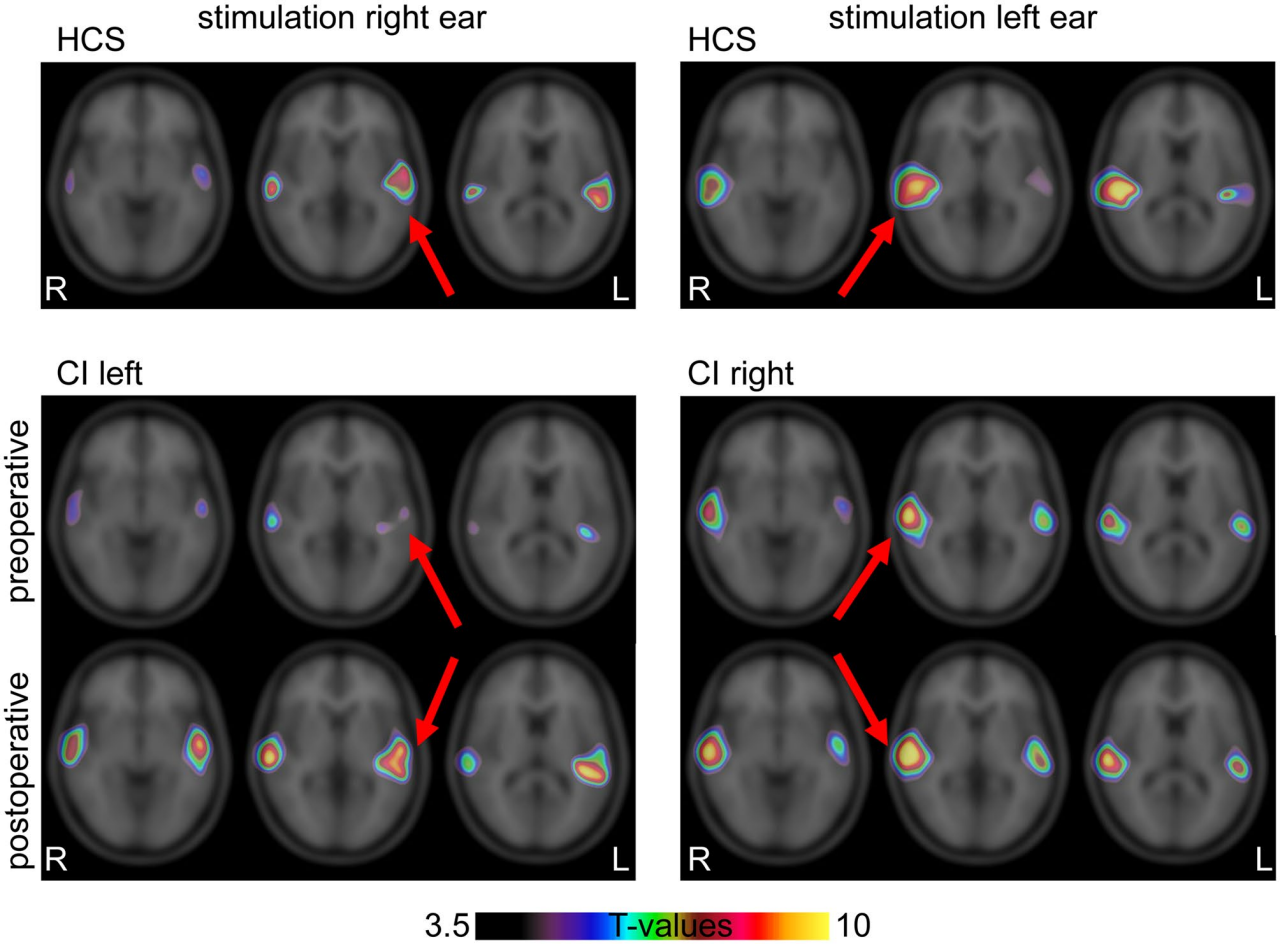
Overlaid statistical parametric for the four groups overlaid on the T1 MNI Template. All results are displayed with a common color scale showing the effect of stimulation. The effect of increased postoperative activity of the auditory signal can be seen in the brain on both sides, more pronounced on the contralateral side of the stimulation (red arrows)

To perform region of interest analysis, the perfusion signal was derived from the significant regions of the voxel-wise analysis with SPM12 (see Fig. 2). To define regions of interest, a cut-off of *t* = 6 for all conditions was taken based on the respective parametric maps (*t*-values) from the SPM analysis. As the results of the different groups were not identical in extension of activity, the intersection outline of the significant contralateral activation in healthy and post-CI patients was taken. These regions of interest (left and right) were also limited to brain outlines using the isocontour option in PMOD. The two regions—one for left site activation with right-sided stimulation and one for right site activation with left-sided activation—were applied on all normalized images and the results coded as either ipsilateral or contralateral.

For the statistical analyses, values from the region of interest analysis were calculated as K1/cerebellum activity (i.e., stimulation minus baseline condition) for each participant on the ipsilateral and contralateral side. To compare the results with a recent publication [32] an asymmetry index (AI) = (Contralateral − Ipsilateral)/(Contralateral + Ipsilateral) was used. Positive values show contralateral neuronal preponderance with increased laterality at higher values. Conversely, negative values indicate ipsilateral neuronal preponderance. The Mann-Whitney *U* test was used to assess group differences between healthy and patient group before and after CI implantation. Repeated measures within the patient group only was not feasible due to low power. Exact significance *p* values for two-sided analysis and *U* values were shown. The following significance levels were used: *p* < 0.05 corresponded to significant (*), *p* < 0.01 to very significant (**), and *p* < 0.001 to highly significant (***). Statistics were performed with SPSS 26 (Statistical Packages for Social Sciences, version 26.0, SPSS Inc., Chicago, IL, USA).

## Results

### Participants

Eight patients (three women) were included in this analysis. Mean age was 44.1 years (range 27.8–56.2, SD = 9.0). The cause of SSD was SSNHL in all cases with no relevant improvement after standard steroid therapy. Inclusion into this study took place only after completed diagnosis and treatment. In addition, all patients were evaluated by MRI before inclusion as defined in the study protocol. The mean duration of SSD before the inclusion date was 1.3 years (range 0.5–4.3, SD = 1.2) and before CI implantation 1.7 years (range 0.8–4.6, SD = 1.2). Four patients had a left-sided and four a right-sided deafness. The mean hearing threshold (mean of 125, 250, 500, 1 k, 2 k, 4 k, 6 k, 8 k Hz) of the SSD side was 73.2 dB (range 51.9–102.5, SD 16.6) and the one of the normal hearing ear 9.6 dB (range 5–13.1, SD 2.7). All SSD subjects reported tinnitus in the deafened ear with varying degrees of impairment. Surgery was uneventful in all patients. The CI was fitted using behavioral measurements following standard clinical procedures [21]. After cochlear implantation, hearing was measured in the sound field with plugging of the healthy ear. The mean hearing threshold was 30.3 dB (range 18.1–50.3, SD 9.4). With regard to the improvement of speech comprehension in noise, sound localization and tinnitus, the participants showed changes that corresponded to the positive impressions of the effect of CI implantation in SSD from the literature (e.g., [17–25]). For detailed data, we refer to our previous publication [21].

Eight gender- and age-matched HCS had a mean age of 46.6 years (range 30.2–58.8, *SD* = 9.2). The mean hearing threshold (mean of 125, 250, 500, 1 k, 2 k, 4 k, 6 k, 8 k Hz) right was 11.4 dB (range 3.6–15.6, SD 4.0) and left 11.2 dB (range 2.5–16.2, SD 4.4). The HCS did not report any tinnitus. Detailed illustrations of the audiograms can be found in our previous publication [21].

### H215O-PET

The pre-implantation PET scan was done 3.2 months (range 1.3–4.9, SD = 1.26) before CI surgery. The mean period of time between CI activation and the second PET scan was 13.5 months (range 8.1–26.6, SD = 6.8). The mean daily wearing duration of the CI up to the time point of the second PET-CT scan was 11.5 h (range 6.75–14, SD 2.5), but effects of wearing durations on PET signal was not evaluated because of too small numbers. Figure 2 presents the group contrasts of stimulus versus baseline for HCS and patients. The statistical maps were written out with cut-off values of *p* = 0.001 uncorrected for multiple comparisons. For the comparisons within HCS group, the *t* value cutoff was 3.6. For the patient group with the additional condition of pre- and post-operation time points, the cut-off was *t* = 3.3. For display purpose, the maps were overlaid on the T1 template with a common *t* value range from 3.5 to 10. The SPM12 results are summarized in Table 1.

**Table 1:** Results of statistical parametric mapping per group analysis

**Group**	**Region (superior temporal gyros encomp. BA 42, 22, 41)**	**MNI coordinates of cluster (x/ y/ z)**	**Peak** ***t*- value**	**kE (cluster level)**	***p*** **FWE-corr**	***p*** **FWE-corr Cluster level**
HCS left	Right side (contralateral)	54/−26/8	11.82	6341	0.000	0.000
	Left side (ipsilateral)	−42/−30/8	8.27	2214	0.005	0.008
HCS right	Left side (contralateral)	−54/−38/10	9.56	3075	0.001	0.004
	Right side (ipsilateral)	62/−26/2	9.42	1554	0.001	0.044
Patient left Preoperative	Right side (contralateral)	64/−18/0	10.30	5625	0.000	0.001
	Left side (ipsilateral)	−58/−26/4	7.68	3284	0.000	0.012
Patient left Postoperative	Right side (contralateral)	64/−18/0	11.99	7013	0.000	0.000
	Left side (ipsilateral)	−56/−28/6	8.23	4001	0.000	0.006
Patient right stimulus Preoperative	Left side (contralateral)	−38/−32/8	6.65	2551	0.000	0.018
	Right side (ipsilateral)	64/−24/0	6.69	2413	0.000	0.022
Patient right stimulus Postoperative	Left side (contralateral)	−44/−34/8	10.09	6594	0.000	0.000
	Right side (ipsilateral)	64/−22/0	10.21	4493	0.000	0.002

These results were used for the regions of interested definition as described in the "Analysis" section. To focus on common activation, the intersection of both groups was taken as described above. The regions showing most differences in pre- and post-CI are indicated by red arrows in Fig. 2.

The right-sided stimulation in the HCS showed bilateral activity with contralateral predominance of the Heschl’s Gyrus region and posterior part of superior temporal gyrus (encompassing parts of Brodmann-Area (BA) 42, BA22 and BA 41). In Table 1, the coordinates and statistical values for each activation on the right or left side are displayed per group and time point. Left-sided stimulation showed predominantly contralateral activity of the same region in HCS. Before CI, patients with right acoustic stimulation (CI left side) demonstrated a low activity in both regions with even lower activity on the contralateral left side. After CI use, these patients showed a similar neuronal activity as the HCS with a bilateral but predominantly contralateral activity of the same regions.

Direct comparisons between pre- and post-CI for the patients with left-sided acoustic stimulation (CI right side) revealed a small difference in right inferior temporal gyrus (MNIxyz = 58 −56 −12, puncorr = 0.000, *t* = 5.70, *p*FWE-corr = 0.006) and a small difference of bilateral activations in the region of mediotemporal cortex/ventral diencephalon (MNIxyz = 30 −16, −8, *p* uncorr = 0.000, *t* = 3.94 and MNIxyz = −14 −26 −8, *p* uncorr = 0.000, *t* = 4.24), primarily due to signals in the stimulation condition.

The direct comparisons between stimulation and control condition in both sessions and also in the healthy control group revealed primarily a difference of signal intensity between the comparisons. The location of activation remained the same and consistent, only the extension of significant voxels around the center of activation differed. Differences can therefore be assumed to be primarily relevant with regard to activation level and not anatomically.

**Fig. 3:**
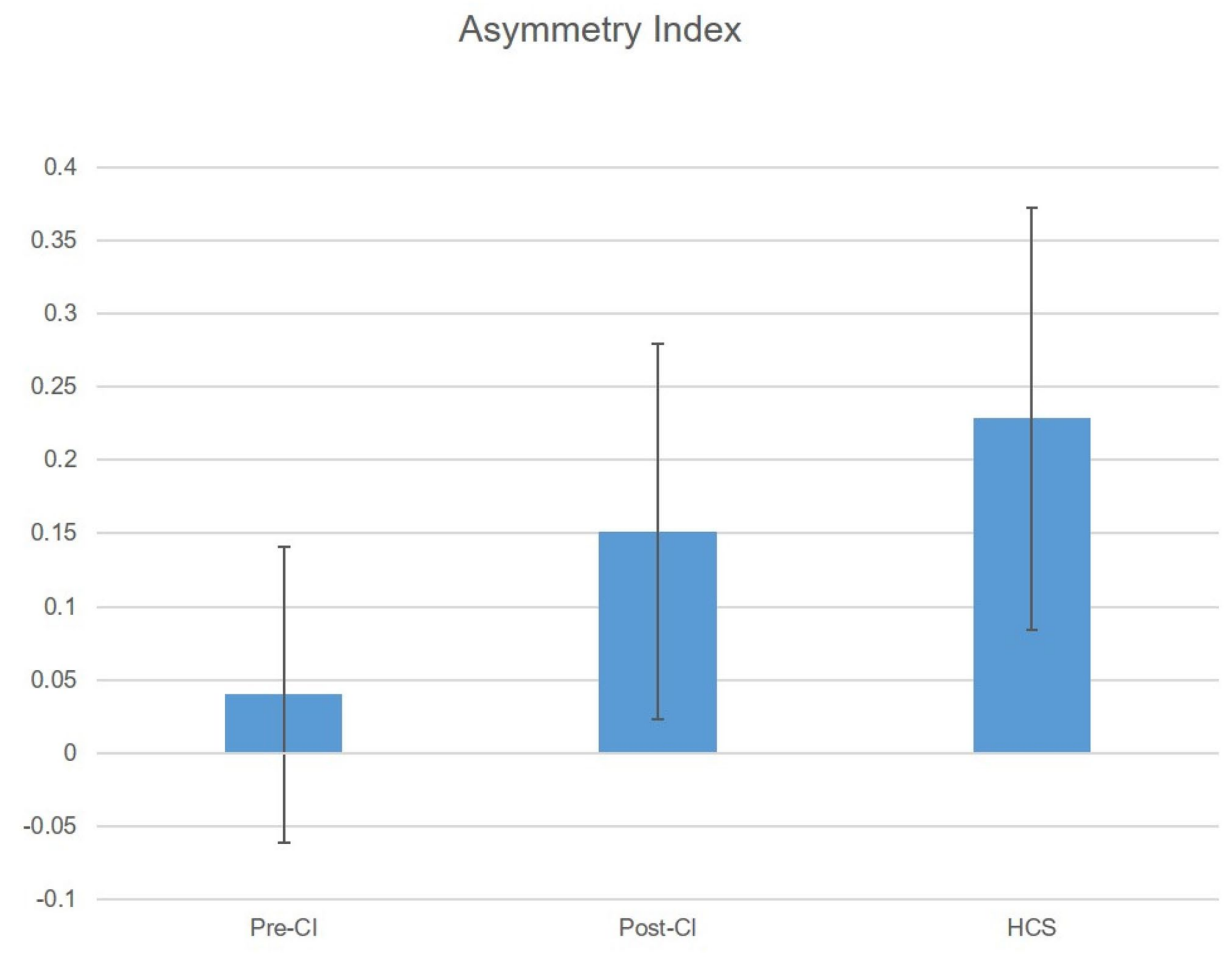
Comparison of the asymmetry index (AI) between pre- and post-CI condition with HCS. Mann-Whitney *U* test between pre-CI and HCS (**p < 0.01), between post-CI and HCS (non-significant)

Higher positive values of the AI indicate more pronounced contralateral neuronal activation. The group AI including all eight patients was significantly smaller than the HCS-group AI before CI (Mann-Whitney *U* test exact two-sided *p* = 0.002, *U* = 4, standard error 9.5, standardized test statistic −2.94, mean rank HCS = 12, mean rank patients = 5) (Fig. 3). The patient group AI increased after cochlear implantation and the difference between the HCS group and the patient group was no more significant (exact two-sided *p* = 0.195, *U* = 19, standard error 9.5, standardized test statistic −1.365, mean rank HCS = 10.13, mean rank patients = 6.88) (Fig. 3).

**Fig. 4:**
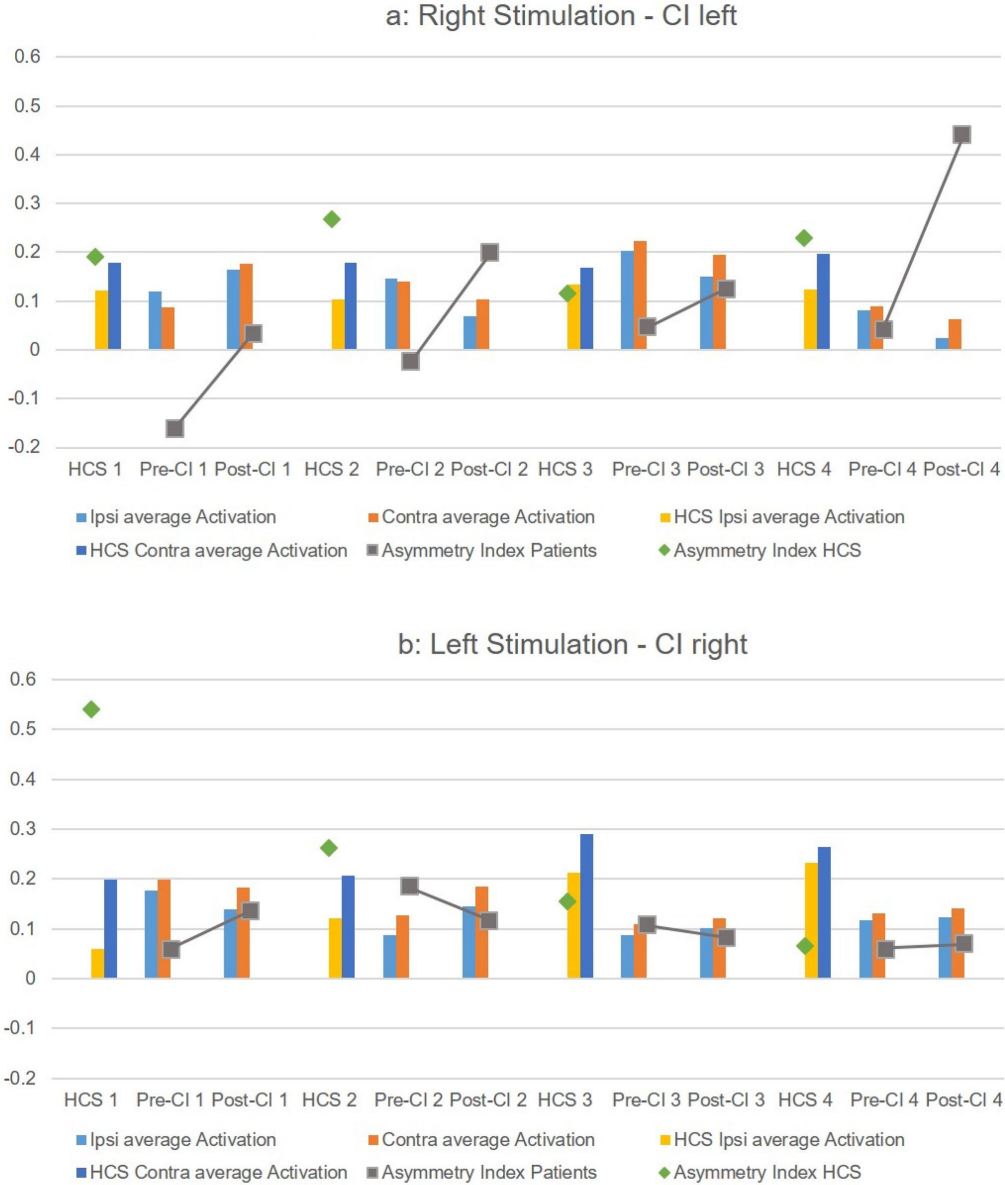
Contra- and ipsilateral activity and the AI for individual patient with corresponding HCS

Figure 4 summarizes the individual contra- and ipsilateral activities and the AI for all patients together with their matched HCS. A stronger contralateral activation is evident for patients with right-sided stimulation (CI left) than for patients with left-sided stimulation (CI right). The AI of all four patients with right-sided stimulation increased clearly after CI, while the AI decreased in two patients with left-sided stimulation. However, the activity itself increased moderately in these two patients.

## Discussion

In this [15O]H2O-PET study, we demonstrated that acoustic stimulation of patients with SSD evoked more symmetric patterns of activation in the auditory cortices than those of a matched group of subjects with normal hearing on both sides (Figs. 2, 3 and 4). Moreover, we showed that the use of a CI reversed these changes towards more normal patterns. When patients with SSD used a CI on their deaf ear, they developed a more asymmetric activity of the auditory cortices to an unchanged acoustic stimulation of their normal hearing ear. The patterns with CI became more similar to those of the matched HCS. These findings support the view that CI in SSD affects the entire auditory processing permanently. Regaining hearing on the deafened side induced higher neuroplastic changes, including processing by the contralateral hearing ear. It may be assumed that at least part of the salutary effects of CI on hearing and on tinnitus in SSD is related to such changes.

It has been shown in many clinical studies that CI in SSD can improve speech understanding in noise, the localization of sound, and that it can suppress tinnitus (e.g., [17–25]). Imaging studies of neuronal plasticity induced by CI are more difficult to obtain than auditory studies. Even though ongoing developments on CI devices may improve compatibility with MRI examinations [2], artifacts caused by the case and the magnet will remain, affecting in particular the area of the temporal cortex. Examining the auditory cortex as the primary region of interest by fMRI may therefore continue to be problematic.

Our functional imaging study investigating neuroplastic changes after CI in SSD was performed by [15O]H2O-PET. The [15O]H2O-PET examined changes of the rCBF in the auditory cortices during acoustic stimulation compared to a baseline recording without stimulation. Compared to other imaging techniques used to examine the effects of SSD such as fMRI [2–11], EEG [12, 13], or MEG [14], [15O]H2O-PET can only examine relatively slow changes in minutes with relatively limited resolution of 4–7 mm FWHM (full width at half maximum). Only rCBF and not direct neuronal activity is measured. This together with the limited resolution and partial volume effects leads to images representing not only the AC enriched during acoustic stimulation, but also surrounding structures. This limitation seems acceptable since conditions were similar for all participants and the focus was on changes. Within these limitations, we found a reduced hemispheric asymmetry of the auditory cortices in unilateral deafness. However, it must be assumed that other brain areas also involved in neuroplastic changes due to SSD were not visualized with the [15O]H2O-PET technique, possibly also due to the small sample size of our patient group.

A recent [15O]H2O-PET study [32] investigated cortical plasticity after cochlear implantation in asymmetric hearing loss (AHL) with similarities to ours. The size of the study was similar with an inclusion of ten patients with unilateral CI after postlingual AHL and ten controls with normal hearing. In contrast to our study, the patients were examined once only with four different conditions of acoustic stimulation (baseline, bilateral, right, left). Results of this [15O]H2O-PET study after CI implantation [32] were compared to those of a previous study of the same research group [8], which examined acoustic stimulation of the normal hearing ear in SSD patients with fMRI. A normalization of interhemispheric asymmetry was demonstrated. We confirm this finding with our prospectively collected data within the same patient group before and after CI. Karoui et al. investigated also stimulation by CI and found a tendency of contralateral predominance, but without statistical significance [32]. The AI of all test conditions was not significantly different in comparison to the control group [32]. Notably, contralateral dominance during stimulation of the non-implanted ear correlated with performance of sound localization [32].

Even though laterality of the postlingual SSD seems to have an influence on the patterns of adaptive changes [27, 35–41], our sample of four right- and four left-sided SSD was too small to investigate laterality differences. Nevertheless, the four patients with a left-sided CI had remarkable improvements of their AI in comparison to the four right-sided patients. It seems possible that they benefited more from their CI. Laterality differences may be attributed to the distinct lateralization asymmetries in normal binaural auditory central processing. Larger samples would be needed to investigate this further.

A major strength of our prospective study was the direct visualization of neuroplastic changes with [15O]H2O-PET before and after CI in the same subjects. These imaging methods were not influenced by the implantation of a magnet. Since the CI was not stimulated during examination, artifacts such as described, for example, in the CEAP responses were avoided [26].

There are several limitations to our study. The sample size was too small to allow for statements about right-left differences. Moreover, the use of radioactive tracers in [15O]H2O-PET examinations limited the number of examinations per subject. For this reason, our study design focused on postoperative measurements of the normal hearing ear only, and not on CI stimulation. In addition, the [15O]H2O tracer with a half-life of 2 min must be produced on the examination site, which causes waiting periods of 10 min between scans. Consequently, the total examination time was approximately 1.5 h in the scanner. Patients were reminded of the next examination before a scan, but fatigue with effects on the results of the cortical activities cannot be excluded. Unfortunately, additional unforeseen and prolonged technical problems with the onsite radionuclide production by a cyclotron necessitated deviation from the protocol and a wider time range of postoperative examinations than initially planned.

In summary, our [15O]H2O-PET study showed more symmetrical rCBF in the AC when the healthy ear was stimulated in SSD than in HCS. Secondly, we were able to show a reversion of these neuroplastic changes to more normal patterns in SSD patients after the regular use of a CI. This objective finding complements the well-established positive subjective effects of CI in SSD. Subjective benefits include improved speech understanding in noise, improved sound localization, reduced tinnitus perception, better general hearing ability, and improved quality of life (e.g., [17–25, 32]). In view of the fact that CI in SSD is still not reimbursed in several countries [2], further studies on neuroplastic changes caused by CI in SSD with larger numbers of cases are desirable, such as with PET-CT, EEG, or with other techniques. Such studies could also help to better predict the outcome of CI in SSD and determine the period of deafness in which a reversion of neuroplastic changes by CI can still be expected.
